# Ginger and the beetle: Evidence of primitive pollination system in a Himalayan endemic alpine ginger (*Roscoea alpina*, Zingiberaceae)

**DOI:** 10.1371/journal.pone.0180460

**Published:** 2017-07-19

**Authors:** Babu Ram Paudel, Mani Shrestha, Adrian G. Dyer, Qing-Jun Li

**Affiliations:** 1 Key laboratory of Tropical Forest Ecology, Xishuangbanna Tropical Botanical Garden, Chinese Academy of Sciences, Yunnan, China; 2 University of Chinese Academy of Sciences, Beijing, China; 3 Department of Botany, Prithvi Narayan Campus, Tribhuvan University, Pokhara, Nepal; 4 School of Media and Communication, RMIT University, Melbourne, Victoria, Australia; 5 Faculty of Information Technology, Monash University, Melbourne, Victoria, Australia; 6 Laboratory of Ecology and Evolutionary Biology, State Key Laboratory for Conservation and Utilization of Bio-Resources in Yunnan, Yunnan University, Kunming, Yunnan, China; Indian Institute of Science, INDIA

## Abstract

The Himalayan endemic alpine genus *Roscoea*, like other members of ginger family, exhibits the combination of floral traits that would fit pollination by long distant foragers such as bees, birds or flies. We studied the pollination biology of *Roscoea alpina*, observed potential floral visitors and determined their foraging behaviour, visitation frequency and pollination efficiency, to seek evidence in support of the pollination syndrome hypothesis. We also measured the floral spectra of *R*. *alpina* flowers to evaluate if signals fit with the currently known framework for observed floral visitors. We found that *R*. *alpina* have autonomous selfing and pollinator-mediated crossing, but lack apomixis. We observed that a beetle (*Mylabris* sp.), and a moth (*Macroglossum nycteris*) visit the flowers of *R*. *alpina* for pollen and nectar feeding respectively. Our field observations, the stigmatic pollen count and fruit set data indicated that the visit by the beetle was legitimate, while that of the moth was illegitimate. Emasculated flowers visited by beetles set as many fruits and seeds/fruit as auto-selfed and naturally pollinated flowers, while emasculated flowers excluded from beetle visits did not set fruit and seed; indicating that a single visit of a beetle to the flowers of *R*. *alpina* can facilitate pollination. We found that flower spectral signal of *R*. *alpina* does not fit typical spectra previously reported for beetle or bee-visited flowers. Our results suggest that, to ensure reproductive success in alpine habitat, *R*. *alpina* has evolved autonomous selfing as a predominant mode of reproduction, while beetle pollination would promote genetic diversity of this plant species. The visitation of beetles to the flowers of *R*. *alpina*, despite floral signal mismatch with the classically associated beetle vision, suggests that a different visual processing may operate in this plant-pollinator interaction at high altitudes.

## Introduction

Plant-pollinator interactions form complex relationships that have been intensively studied for over a century to understand the evolution of angiosperms [1–7]. Evidence from the cretaceous period fossils indicates that beetles were likely pollinators of early angiosperms [8–11]. To date beetle pollination is mainly reported from the Mediterranean [12] and African regions [13–15], and there is value in understanding how this may operate in other parts of the world. The Nepalese Himalayan Mountains provide a unique avenue to conduct research on plant-pollinator interactions because of the steep gradient meaning sub-tropical to sub-alpine conditions can exist in close proximity, potentially giving insights into how spatial and temporal distributions of plants may be influenced by climatic conditions [16,17]. So far relatively few studies have carefully quantified the pollination systems that exist for Himalayan plants, and this work suggests the presence of specialized plant-pollinator interactions [18,19].

Gingers (family Zingiberaceae) with about 1300 species in 52 genera are one of the earliest angiosperms that evolved in the Cretaceous with a broad tropical distribution [20,21]. Following the late cretaceous diversification from the ancestral distribution center, gingers underwent tremendous radiation which resulted in many varieties of flowers within the family [20,22]. Such highly diverse flowers within the family are thought to have been the consequences of coevolution with their prime pollinators [23]. Despite apparent diversities, most members of the family have zygomorphic flowers with specialized long corolla tube, traits consistent with insect pollination [22]. Congruent to the floral syndromes, most members of the family are pollinated by long distant foragers such as bees, birds and/or flies that mostly forage on the flowers to collect nectar [18,22,23].

*Roscoea* with 22 species is a Himalayan endemic alpine genus of the predominately tropical family Zingiberaceae [24,25]. The unusual distribution and recent evolution of genus *Roscoea* relative to other members of the family is the consequences of the uplift of the Himalayas subsequent to the collision of the Indian plate with Eurasian plate [26]. Like other plants of the family, flowers of genus *Roscoea* contain nectar as a reward, indicating pollination by nectar feeders such as bees, birds, flies, moths etc. [18]. Moreover, specific floral traits of *Roscoea* such as wide labellum, long corolla tube with nectar, flexible anther, nectar source located away from the reproductive organs, and absence of fragrance suggests adaptation toward long-tongued insects for pollination success [24,27]. This prediction is supported by the historical observations and recent findings in *R*. *purpurea* [18,19,28,29].

In the current study, we investigated the pollination biology of *R*. *alpina*, to test for potential evidence supporting a pollination syndrome. We hypothesized that consistent to the closely related *R*. *purpurea* [18], *R*. *alpina* might involve highly specialized mutualism with long-tongued insects to enable pollination. Surprisingly, however, our observations revealed that a beetle (*Mylabris sp*.) and a moth (*Macroglossum nycteris*) were the only observed floral visitors of *R*. *alpina*. We subsequently focused on the following questions: (i) How does pollination occur in *R*. *alpina*? (ii) Do the observed visitors (beetle and/or moth) act as the effective pollinator of *R*. *alpina*? (iii) How might the floral spectral signal of *R*. *alpina* fit with the currently known framework for beetle and moth vision?

## Materials and methods

### Ethics statement

We obtained general permission from local community forestry users groups, local governmental bodies (Village Development Committee) and Annapurna Conservation Area Project to conduct the research. None of our model species were regarded as within a threat category, and ethics is not required for research of insect observational studies.

### Study species

*Roscoea alpina* is a true alpine species within the genus and is distributed between the elevations of 2130 m to 4270 m in the Himalayan regions (from Kashmir in the west through Punjab, Himachal Pradesh-India, Nepal, Tibet, Sikkim and Bhutan in the east) [24]. It grows either in open meadows or under the canopy of *Rhododendron*, *Pinus*, and/or *Betula* forest. It is perhaps the smallest species amongst the Himalayan *Roscoea*. The erect pseudostem that grows out from the underground rhizome may produce up to four linear, broadly elliptic or lanceolate leaves. The first leaf is slightly auriculate and widest at the base while the rest of the leaves are widest at the middle. Flowering occurs from the end of May to the end of July. *R*. *alpina* produces single inflorescence and only one flower of the inflorescence open at a given time, although an inflorescence bears up to 5 flowers. Flowers are without exerted peduncle and have previously reported pinkish to white flowers, colour characteristics, considering human perception [24].

### Study sites

**Fig 1:**
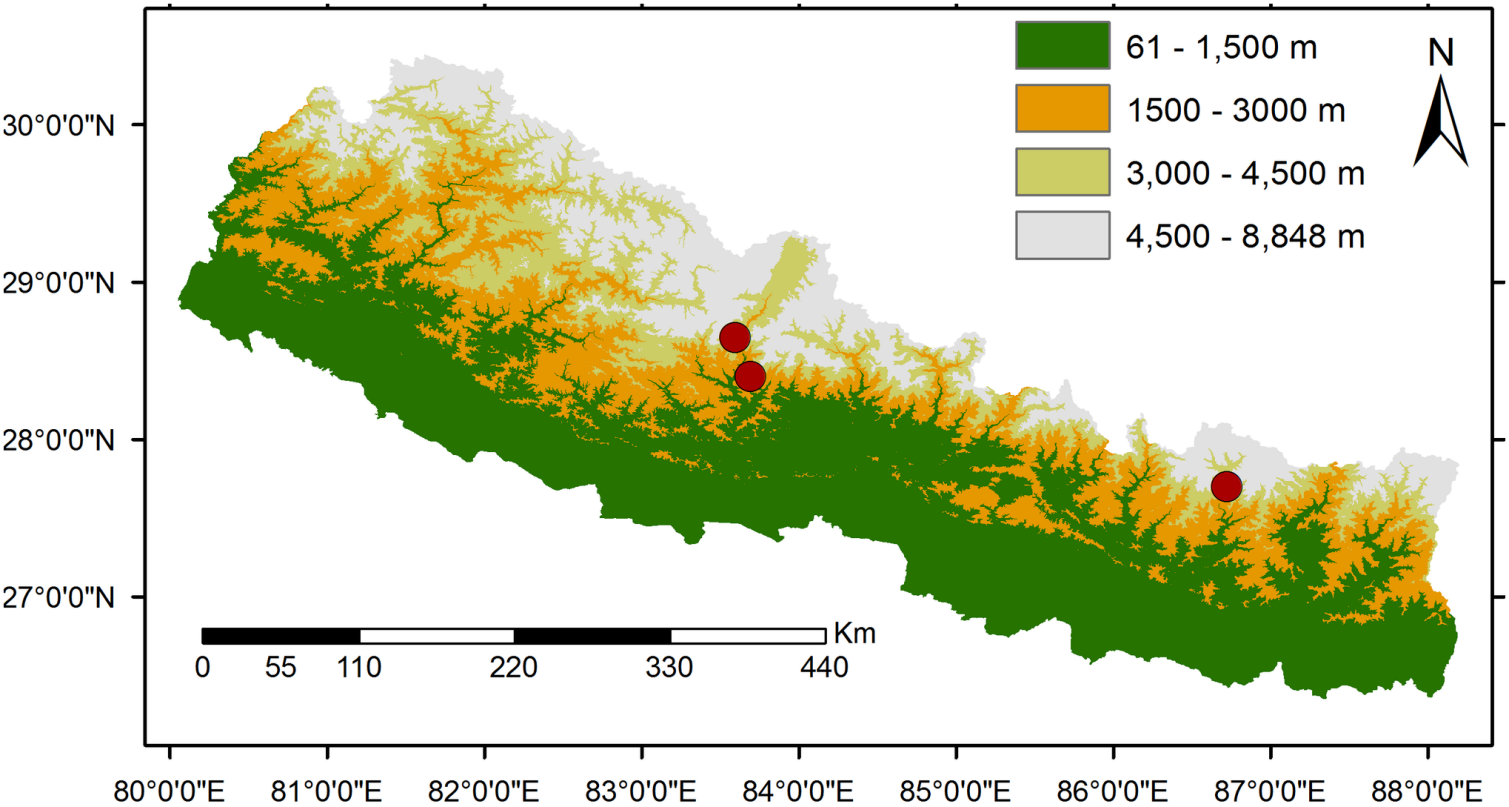
Map of study sites and experimental area. Red circles represent the study sites.

We studied the floral biology and observed the floral visitors of *R*. *alpina* at three different sites; Lete (28°38', 83°35', 2527 m), Mustang; Poonhill (28°24', 83°41'', 3206 m), Myagdi; and Chheplung (27°41', 86°43', 2716 m), Solukhumbu, Nepal (Fig 1). The vegetation at all sites comprises of the mixed forest of *Rhododendron* and *Pinus*. All sites experience subalpine climate and have cool weather around the year, with light rainfall/monsoon in summer (June-August) and snowfall in winter (November-February) (Pers. obs BRP). The temperature of Poonhill ranges from -3° to 18°c in summer time [30].

### Floral biology

We recorded the floral biology of *R*. *alpina* at three sites (Fig 1), for two consecutive years (2015–2016) from early to Mid-June. At each site, we recorded the number of flowers per inflorescence, floral longevity, time of anthesis, anther dehiscence and stigma receptivity of *R*. *alpina* by direct observation. Floral traits (corolla tube length, anther’s length, length of ovary, number of pollen grains per flower, number of ovules per ovary and pollen-ovule ratio) were measured from the freshly opened flowers (n = 20 for each trait) following the method of Paudel et al. [18]. To test if floral traits in *R*. *alpina* vary spatially, we analyzed the differences in floral traits among the three sites using a one-way ANOVA. To find the stigma receptivity, we used Dimethylthiazol-diphenyl-tetrazolium bromide (MTT) to test for the presence of dehydrogenase on the stigma following the method used by Rodriguez-Riano and Dafni [31], Wang et al [32] and Fan and Li [33]. If the stigma turns dark purple–brown with MTT it indicates the presence of dehydrogenase on stigma, suggesting the stigma was receptive [31–33].

**Fig 2:**
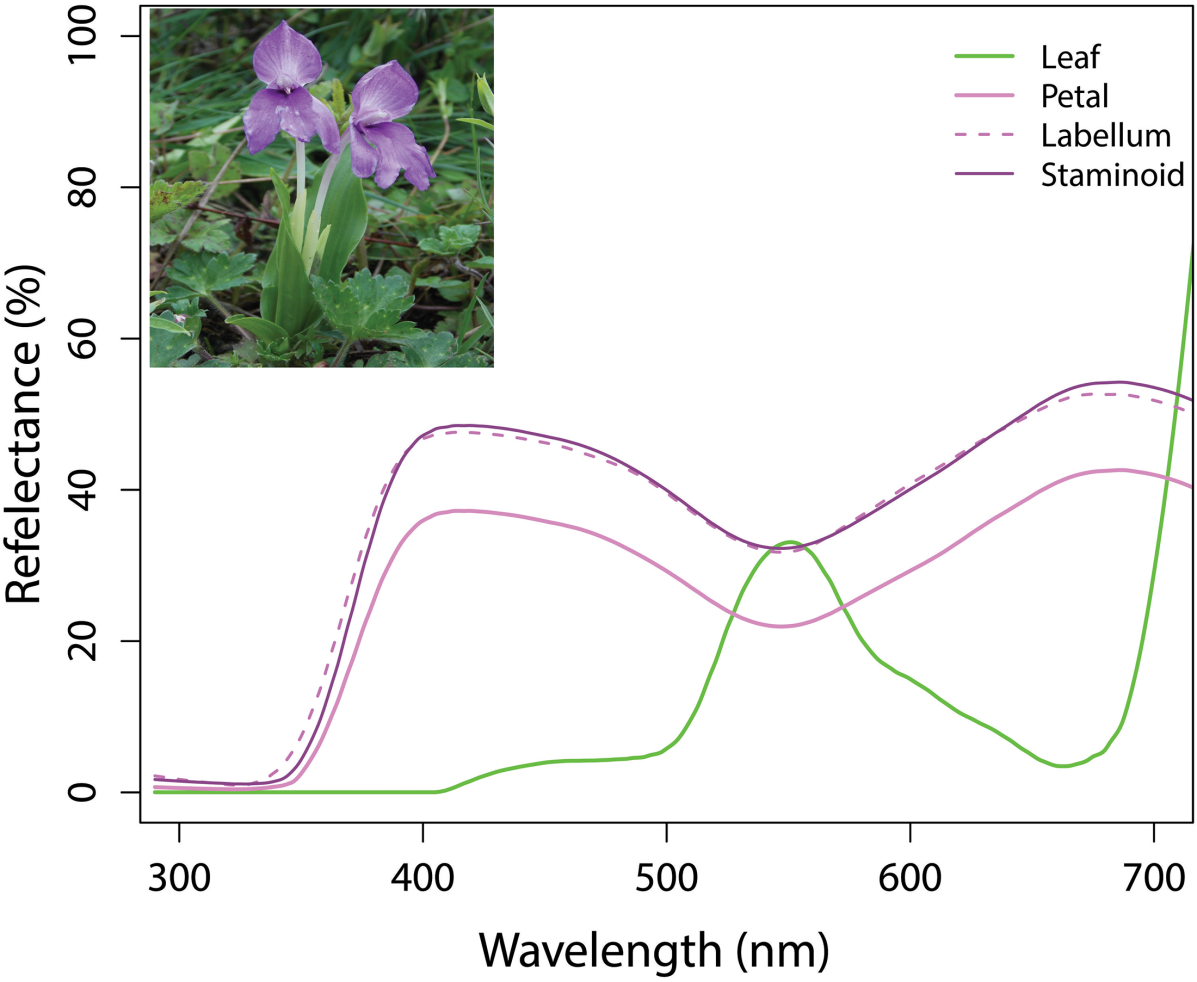
Reflectance spectra of different floral parts of *R*. *alpina*.

To understand plant-pollinator interactions, it is important to consider the different visual capabilities of various flower visitors and reliable measurements of flower spectra [34]. We thus measured the floral reflectance of *R*. *alpina* (at Poonhill) to provide initial insights into whether flower spectra might play a role in signal evolution for this species. Specifically, recent work [35] shows that in the absence of mainstream pollinators that are common in most floral communities, flowers from remote Macquarie Island deep in the southern ocean are a dull cream-green colour, and lack any sharp change in reflectance at wavelengths less than about 420nm. We thus used this as a comparison point for recorded spectra from *R*. *alpina*. We used an OCEAN OPTICS spectrophotometer (USB2000+, Ocean Optics Inc., Dunedin, FL, USA, 2011) with a PX-2 pulsed xenon light source attached to a computer running SPECTRA SUITE software and measured multiple individuals flowers from 300–700 nm wavelength following methods described in Chittka and Menzel [36], Dyer et al. [37] and Shrestha et al. [16]. We used three replica from different flower for each floral parts to measure the reflectance spectra. We provided the raw spectra in Fig 2 for the future analysis, as currently the vision system of the visitor beetle we observed is not studied. We consider the importance of floral colour and beetle vision in the discussion section below to promote future work on this understudied topic.

### Observation of pollinators

We observed the floral visitors of *R*. *alpina* at three sites across central Nepal (Fig 1), from early to Mid-June (2015 and 2016) for a total of 208 hours. The observations were made from 7:00 AM to 19:00 PM for diurnal visitors and from 20:00 to 22:00 PM for nocturnal visitors. We repeated the observations for three days at each site. To identify the legitimate pollinator of *R*. *alpina* and to estimate their visitation frequency, we made three independent plots (10 x 10 m) at the distance of more than 100 m and observed all floral visitors of *R*. *alpina* within the plots. Foraging behaviour of the visitors (legitimate or illegitimate) was recorded by direct observation. The visit was considered legitimate if the visitor’s body touched the reproductive parts of a flower. We estimated the visitation frequencies of the floral visitors in terms of numbers of visit flower^-1^hour^-1^. To test if visitation frequencies differ among the plots and between the years, we analyzed the data using two way ANOVA with plots and years as fixed factors. Finally, visitation frequencies from three independent plots were pooled together and average values of two years were computed. The voucher specimens (plant samples and beetles) were deposited at Annapurna Natural History Museum and Herbarium Center, Prithvi Narayan Campus, Tribhuvan University, Nepal.

### Pollination treatments

Among the three sites selected for the current study, there was low density of flowering individuals of *R*. *alpina* at Chheplung and Lete, thus we were unable to conduct manipulated pollination experiments at these two sites. Hence, we conducted the manipulated pollination treatments only at Poonhill (Fig 1). To understand the natural breeding system of *R*. *alpina*, we conducted six types of pollination treatments: (i) Natural pollination- flowers were tagged and left untouched; (ii) Autonomous selfing- flowers were covered by mesh bags to exclude the pollinator; (iii) Apomixis- flowers were emasculated and covered by fine mesh bags to exclude the pollinators; (iv) Pollinator mediated crossing (emasculated and open)—flowers were emasculated (cleared off the self-pollens by gentle brushing) in the early morning on the first day of anthesis before anther dehisces following the method outlined in Paudel et al.[19] and left exposed to the pollinators; (v) Hand selfing- flowers were manually pollinated using the pollen grains of the same flower; and (vi) Hand crossing- flowers were manually cross-pollinated using the pollen grains from a donor plant lying at least 10 m away, where flowers were emasculated before hand crossing. After 20 days, fruits of each treatment were collected separately and the percentage of fruit set and seed number per fruit of each treatment were evaluated. All the pollination treatments were conducted for two consecutive years (2015 and 2016) to test if the fruit and seed set differed between respective years.

To quantify the capacity of autonomous selfing and its contribution for the natural breeding system of *R*. *alpina*, we analyzed the difference in fruit set percentage and seed number per fruit between autonomous selfing and natural pollination. Similarly, to evaluate the contribution of pollinators for the natural breeding of *R*. *alpina*, we computed the differences in fruit set proportion and seed number per fruit between natural pollination and emasculated-open pollination (pollinator mediated crossing). To test for self-compatibility in *R*. *alpina*, we analyzed the fruit set and seed set differences between hand self-pollinated and hand cross- pollinated flowers. For all these analyses, we used generalized linear models (GLMs) with binary distribution of errors for analyzing the differences in fruit set percentages while we used GLMs with Poisson distribution of errors to estimate the differences in seed number per fruit. We used factorial GLM to test if fruit set and seed set are affected by treatment and year factors.

### Pollinator importance and pollination efficiency

To measure the potential importance and pollination efficiency of a visitor to the flowers of *R*. *alpina*, we covered the matured buds (n = 240) with fine mesh bags until anthesis. Upon anthesis, flowers were emasculated in the early morning before pollinators start to visit the flowers. Those experimental flowers were assigned into three groups (n = 80 for each group), in a counterbalanced random fashion. **(1)** and **(2)** Emasculation and exposure to the pollinators. When a pollinator (either a beetle or a moth) visited the experimental flower assigned to either group 1 or group 2, it was freely allowed to forage. Subsequent to the departure of the pollinator from the flower, the stigma was immediately collected and fixed in 70% ethanol, if the flower was assigned to group 1 while the flower was immediately covered by a fine mesh bag until wilting, if it was assigned in group 2. **(3)** Emasculation and pollinators exclusion- emasculated flowers were left covered by fine mesh bags until wilting. This treatment was designed to exclude the potential pollinators and to check if emasculated flowers set fruit/seed without a pollinator visit. Fruit set by treatments (2) and (3) were collected after 20 days, and percentage of fruit set and seed number per fruit were quantified. Emasculated flowers visited by the moth (n = 40) neither set fruit and seed nor received any pollen grains on their stigma. We repeated the experiment for two consecutive years and found the same result. Thus, we concluded that moths were not a viable pollinator of *R*. *alpina*, and thus moths were not considered for subsequent analysis (see below). The potential importance of a beetle to the flower of *R*. *alpina* was assessed by analyzing the difference in fruit set percentage and seed number per fruit between emasculated-open flowers visited by the beetles (treatment 2) and emasculated flowers that were excluded from the beetle’s visit (treatment 3). We also analyzed the differences in fruit set percentage and seed number per fruit of emasculated-beetle pollinated flowers with natural pollination and autonomous selfing to estimate the potential efficiency of beetle, using GLM with binary and Poisson distribution of errors respectively.

The number of pollen grains deposited on a virgin stigma of *R*. *alpina* by a beetle during a single foraging bout (treatment 1) was counted under the microscope adopting the method outlined in Dafni et al. [38]. For this, we suspended all the pollen grains on ethanol solution by gentle shaking to release all the pollen grains to the ethanol. Then 20 μl of ethanol was taken in a haemocytometer and number of pollen grains present in the ethanol were counted under a microscope. Here, we assumed that pollen grains were uniformly distributed in the ethanol following the method used by Dafni et al. [38], Fan and Li [33] and Paudel et al. [18,19]. To estimate the pollination efficiency index (PEI) of a beetle to the flower of *R*. *alpina*, we used the formula; PEI = visitation frequency × stigmatic pollen deposition following Ne’eman et al.[39]. We used independent sample *t* test to analyze the differences in PEI of a beetle between two years.

## Results

### Floral biology

Flowering in *R*. *alpina* started from the end of May and persisted up to the end of July, with peak blooming from early June to late June. Anthesis occurred in early morning (before 8:00 AM) and soon anthers became ready to dehisce and stigma became receptive. Thus, *R*. *alpina* showed consistency for diurnal pollination. Stigma remained receptive until the complete wilting of flowers. All the floral traits of *R*. *alpina* did not differ significantly across the three study sites (one way ANOVA, P>0.05, Table 1).

**Table 1:** Floral traits (Mean ±SE) of *R*. *alpina* and their variation at three study sites. Result analyzed by a one way ANOVA.

Floral traits	Poonhill	Chheplung	Lete	*F*	*P*
Flower per inflorescence	2.45±0.15	2.75±0.18	2.85±0.17	1.581	0.215
Floral longevity (days)	2.95±0.15	3.1±0.16	3.4±0.14	2.342	0.105
Length of corolla tube (mm)	102.2±2.07	100.85±1.27	98.7±1.70	1.065	0.352
Length of Anther (mm)	5.55±0.19	5.45±0.14	6.05±0.23	3.032	0.056
Length of ovary (mm)	11.55±0.67	10.95±0.42	11.05±0.42	0.397	0.674
No. of pollen grains/flower	11418±622	11744±638	12950±626	1.648	0.201
No. of ovule/flower	58±2	59±2	61±1	1.143	0.326
Pollen-ovule ratio(P/O)	199.78±11.59	199.78±11.33	212.01±6.3	0.413	0.664

### Observation of pollinators

**Fig 3:**
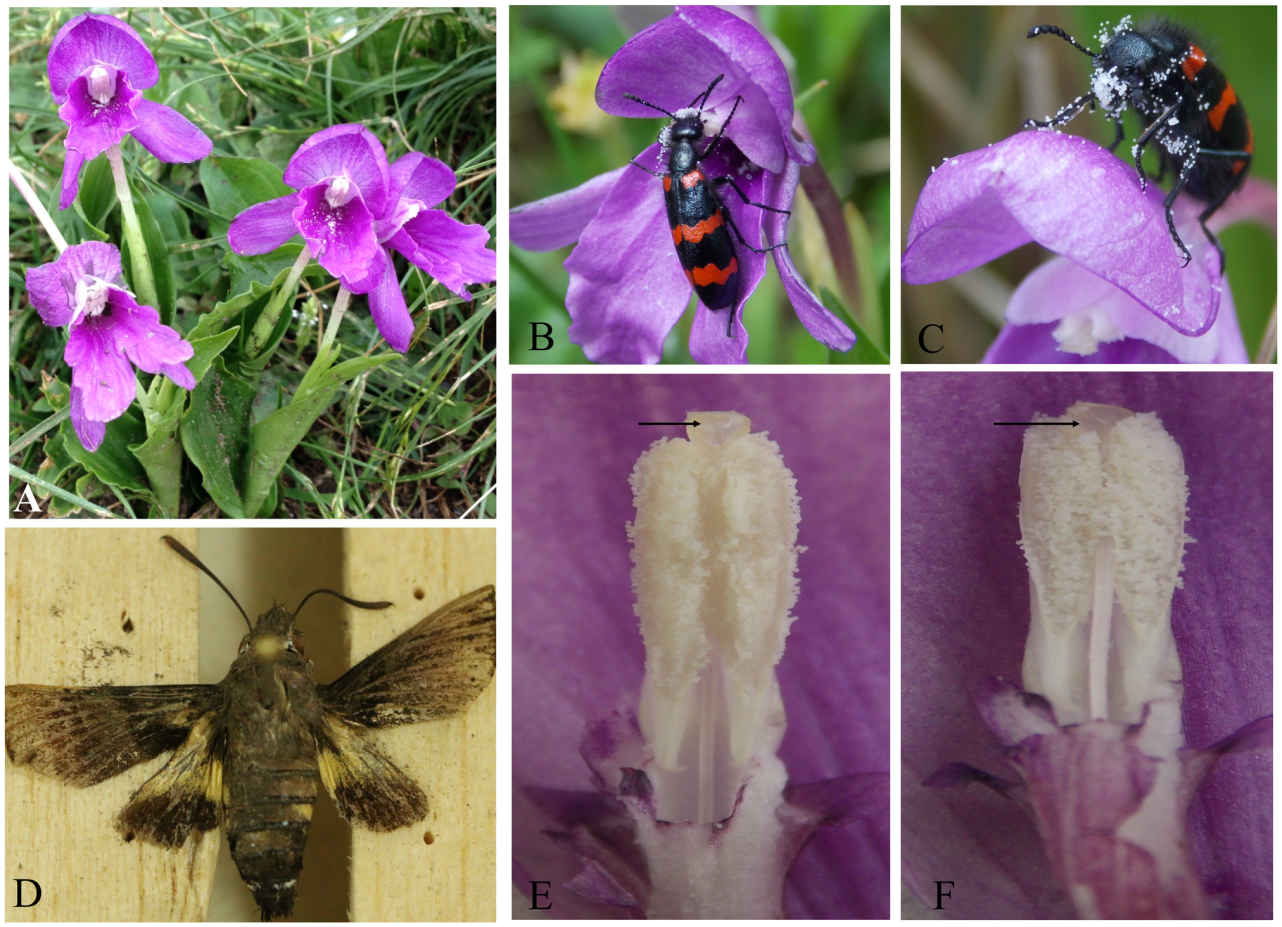
Plant and pollinator. A- Flowering individuals of *R*. *alpina*; B- A beetle (*Mylabris* sp.) feeding on the pollen grains of *R*. *alpina*; C- A beetle resting on the outer part of corolla with thousands of pollen grains attached throughout its body; D- A moth (*Macroglossum nycteris*) caught from a flower of *R*. *alpina* while visiting the flower for nectar feeding; E and F indicate the gradual shrinkage of style to allow self-pollination in *R*. *alpina*. The shrinkage of style occurs ca 2 mm to facilitate self-pollination. E- The position of stigma on the first day, and F- The position of stigma on the third day of flowering.

**Fig 4:**
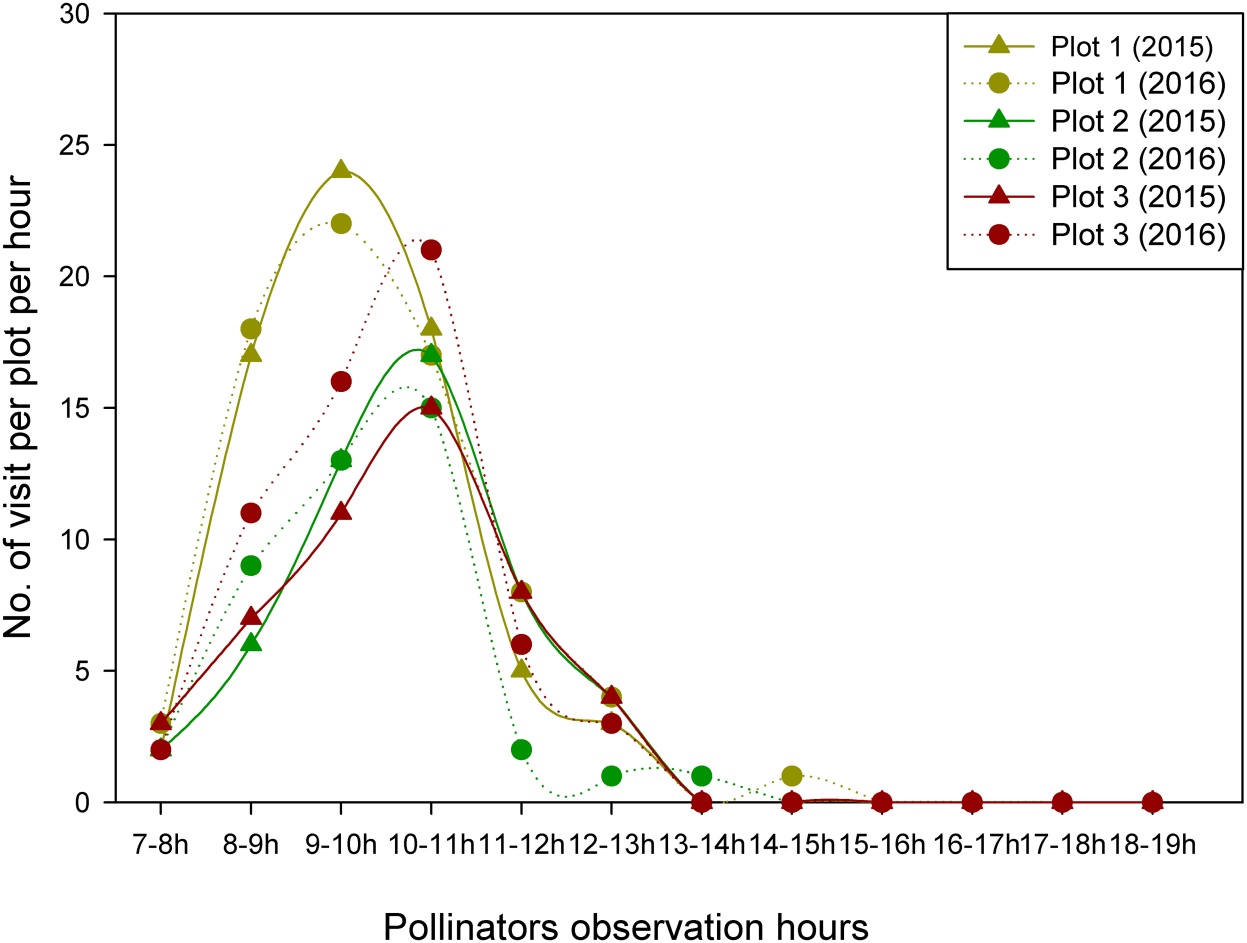
Hourly variation (from 7:00 AM to 19:00 PM) in the abundance of a beetle (*Mylabris sp*.) to the flowers of *R*. *alpina* in 2015 and 2016 at three independent plots at Poonhill.

Our two years of observations during the peak blooming period across the three populations of *R*. *alpina* indicated the scarcity of long distant foragers as the pollination vectors. Due to low density of flowering individuals of *R*. *alpina* at Chheplung and Lete, we did not observe any pollinators visiting the flowers of *R*. *alpina*. However, at Poonhill, a beetle (*Mylabris sp*.) was observed frequently visiting the flowers of *R*. *alpina* to feed on the pollen grains (Fig 3B). During flower foraging, the beetle moved across all parts of the flower, and actively fed on pollen grains, and pollen grains were observed adhered on multiple parts of the beetle (Fig 3B). This action effectively transferred the pollen grains onto the stigma. Thus, a beetle was found as the legitimate pollinator of *R*. *alpina*. Beetles started to visit the flowers of *R*. *alpina* around 8:00 AM when there was abundant sunshine and their activity persisted up to around 14:00 PM (Fig 4). However, beetles did not visit the flowers if the rain was present and their activity was low during periods of cloud cover, suggesting climatic conditions may affect this pollination system.

**Fig 5:**
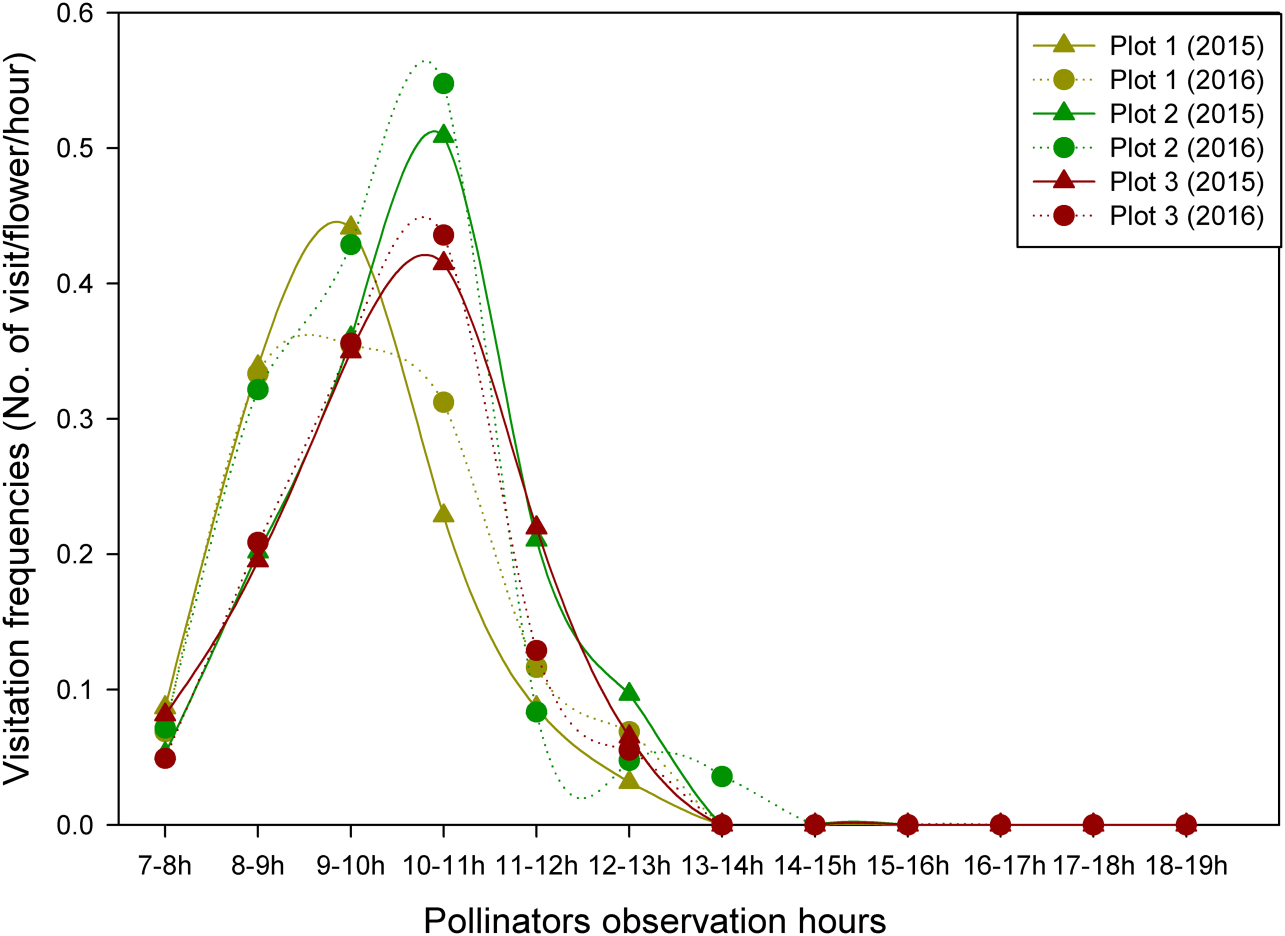
Hourly variation (from 7:00 AM to 19:00 PM) in visitation frequency of a beetle (*Mylabris* sp.) to the flowers of *R*. *alpina* in 2015 and 2016 at three independent plots at Poonhill.

The visits of beetles to the flowers of *R*. *alpina* were not consistent. They either exhibited intraspecies movement (moved within the individuals of *R*. *alpina*) or interspecies movement (moved from the flower of *R*. *alpina* to other plant species that co-flower with *R*. *alpina*). In the year 2015, within 12 hours (from 7:00 AM to 19:00 PM), we observed a total of 154, 237 and 163 visits of beetle to the flowers of *R*. *alpina* at plot1, plot2 and plot3 respectively. Similarly, in the year 2016, within 12 hours (from 7:00 AM to 19:00 PM), we observed a total of 129, 163 and 201 visits of beetle to the flowers of *R*. *alpina* at plot1, plot2 and plot3 respectively. The visitation frequencies of beetles to the flowers of *R*. *alpina* did not differ significantly among plots, between-years and year-plot interactions (two way ANOVA, P>0.05, S1 Table). The visitation frequencies of the beetles were at the peak between 9 to 11 AM, then decreased gradually, and after 14:00 PM beetles did not visit the flowers of *R*. *alpina* (Fig 5). The average visitation frequencies of the beetle, estimated from three independent plots, to the flowers of *R*. *alpina* (observed during the sunny days), were 0.11±0.03 and 0.12±0.03 visits flower^-1^ hour^-1^, for 2015 and 2016 respectively.

At Poonhill, we also observed a few occasional visits of a moth (*Macroglossum nycteris*) to the flowers of *R*. *alpina* for nectar feeding (Fig 3C). Due to rare occasional visits, we were unable to make the reliable quantification of visitation frequency of the moth. During flower foraging, the moth sipped the nectar by hovering over the flowers and very rarely landed on the flower. In either of the situations, the moth did not make legitimate contact with the floral parts of *R*. *alpina* and thus the visit of the moth to the flowers of *R*. *alpina* was illegitimate. We were neither able to measure the amount of nectar present in *R*. *alpina* nor able to calculate the volume of nectar harvested by moth, as the diameter of corolla tube was too small to accommodate the available capillary tube with us in the field.

### Pollination treatments

Among the six pollination treatments, emasculated and bagged flowers did not set fruit and seed indicating the absence of apomixis. However, the rest of the treatments (autonomous selfing, natural, hand crossing, hand selfing and emasculated-open flowers) set fruits and seeds, showing that *R*. *alpina* is self-compatible and endures dual natural breeding system (autonomous self-pollination and pollinator mediated pollination).

**Fig 6:**
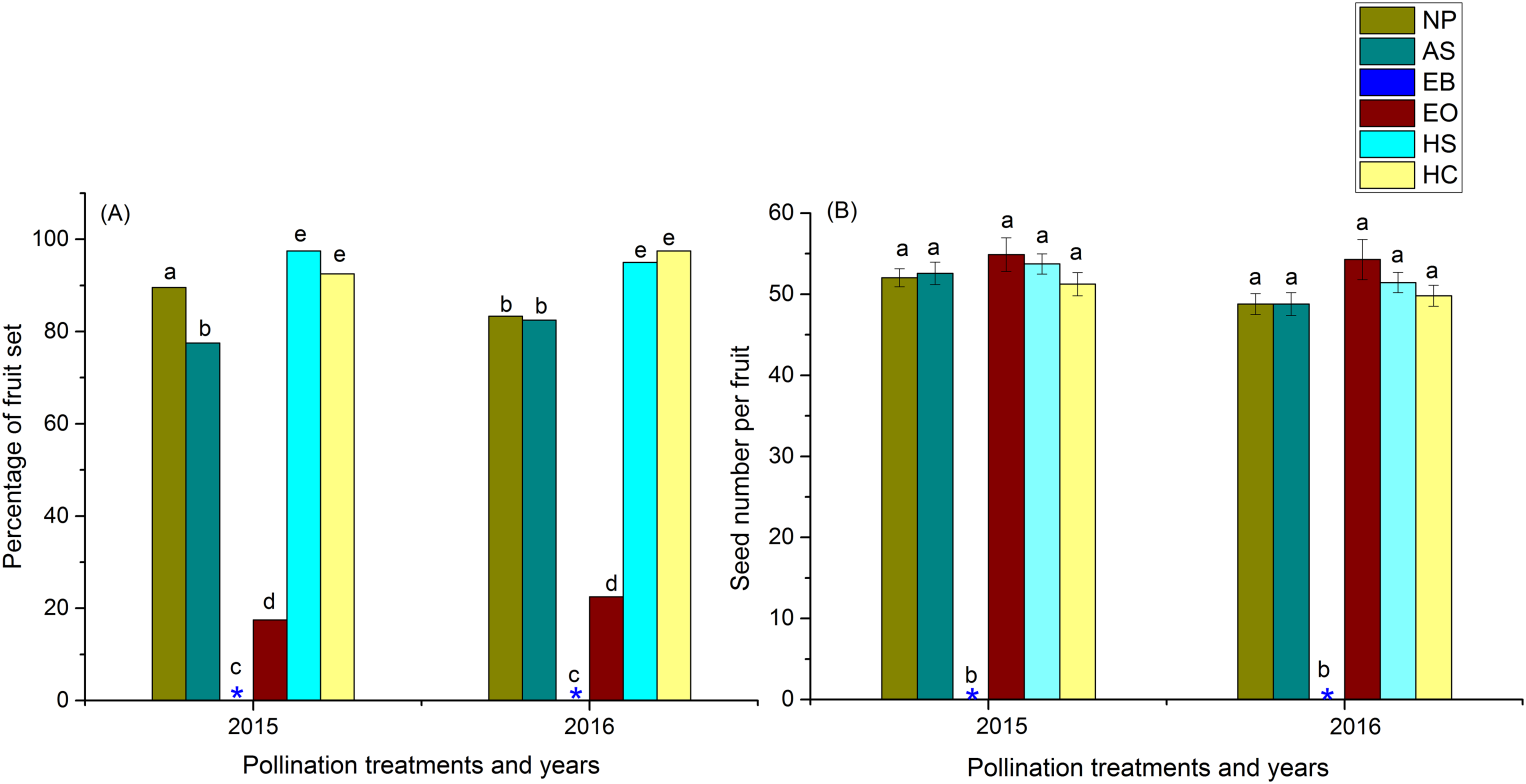
Fruit set percentage and seed number per fruit among different pollination treatments in *R*. *alpina*. Error bars represent the standard error of mean. Different lowercase letters above the bar represent the statistical difference at P<0.05. Asterisk marks in the bar diagram represent zero fruit and seed set. NP = Natural pollination; AS = Autonomous selfing; EB = emasculated- bagged (Apomixis); EO = Emasculated-open pollination; HS = hand self–pollination and HC = Hand cross pollination respectively.

The percentage of fruit set and seed number per fruit between autonomous selfing and natural pollination in *R*. *alpina* did not differ significantly between years, treatments and year-treatment interactions (GLM, P>0.05, S2 Table). These results indicate the high capacity of autonomous selfing in the natural population of *R*. *alpina* and also signify that natural breeding in *R*. *alpina* occurs primarily through autonomous selfing. The emasculated-open flowers set a significantly lower percentage of fruit than natural pollination (Fig 6A), but seed number per fruit between these treatments did not differ significantly between years, treatments and year-treatment interactions (GLM, P>0.05, S3 Table, Fig 6B). The percentage of fruit set and seed number per fruit of hand self-pollinated and hand cross-pollinated flowers in *R*. *alpina* did not differ significantly between years, treatments and year-treatment interactions (GLM, P>0.05, S4 Table). These results show that *R*. *alpina* is fully self-compatible.

### Pollinator importance and pollination efficiency

**Fig 7:**
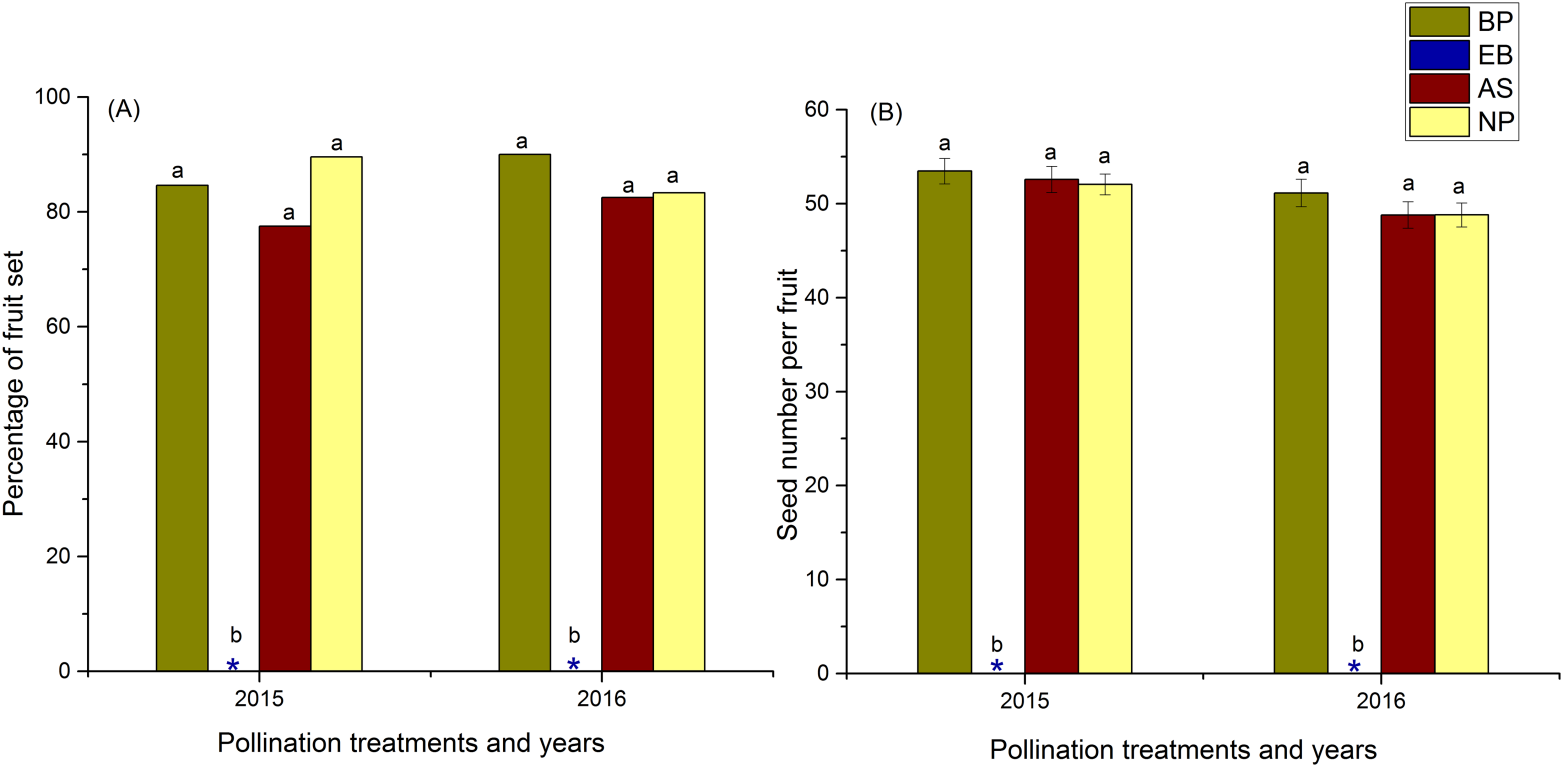
Pollination efficiency and potential importance of a beetle (*Mylabris sp*.) as a pollinator of *R*. *alpina*. Error bars represent the standard error of mean. Different lowercase letters above the bar represent the statistical difference at P<0.05. Asterisk marks in the bar diagram represent zero fruit and seed set. BP = Pollination in emasculated-open flowers after a single visit of a beetle; EB = emasculated- bagged; AS = Autonomous selfing; NP = natural pollination. Emasculated flowers visited by the beetle set as many fruits and seeds as auto-selfed and naturally pollinated flowers while emasculated flowers refrained from beetle visit did not set fruit and seed. This indicates that if a beetle visits a flower, it can affect pollination in *R*. *alpina* as efficiently as autonomous selfing.

We found that the emasculated flowers visited by the beetles set fruits and seeds for both the years. Fruit set and seed production of this treatment did not differ significantly between the years (GLM, P>0.05), indicating that the pollination capacity of the beetles is independent of year factor. A total of 84.6% (n = 39, 1 of the experimental individual was lost,) and 90% (n = 40) of emasculated flowers set fruits in the year 2015 and 2016 respectively. The average number of seeds per fruit was 53.5±1.4 (n = 33) and 51.2±1.5 (n = 36) for the year 2015 and 2016 respectively. While, the emasculated flowers that were excluded from beetle’s visits did not set fruit and seed for either of the years (Fig 7). This result indicates that the beetle (*Mylabris sp*.) is an effective pollinator of *R*. *alpina*. The percentage of fruit set and seed number per fruit in emasculated-beetle pollination, natural pollination and autonomous self-pollination did not differ significantly between years, treatments and year-treatment interactions (GLM, P>0.05, S5 Table, Fig 7). These results indicate that if a beetle visits the flower of *R*. *alpina*, it can contribute, as efficiently as the autonomous selfing, for the female reproductive success of this plant species.

The average number of pollen grains deposited by a beetle on a virgin stigma of *R*. *alpin*a during its single visit was 763 ± 75 and 788 ± 86 in the year 2015 and 2016 respectively. The number of pollen grains deposited by a beetle on the virgin stigma of *R*. *alpina* was several times greater than the number of ovules per flower (Table 1). Pollinator importance index (PEI) values, calculated as the multiplication product of visitation frequency × stigmatic pollen deposition did not differ significantly between the years (*t* test, P>0.05) and were 89.57±23.65 and 94.82±23.78 for 2015 and 2016 respectively. This indicates that an individual beetle during its single visit to a flower can successfully affect pollination in *R*. *alpina*.

The emasculated flowers visited by moths did not set fruit and seed. Similarly, the moth did not deposit any pollen grains on the stigma when it visited the flowers of *R*. *alpina*. These data indicate that the moths do not contribute to the pollination success of *R*. *alpina* though it visits the flowers for nectar feeding.

## Discussion

### Autonomous selfing as a mean of reproductive assurance

Like other species of the family Zingiberaceae, anther and stigma in *R*. *alpina* are spatially separated with stigma lying above the anther. Hence, *R*. *alpina* similar to other Himalayan *Roscoea* species [18] seems to rely on pollination vectors to promote the reproductive success. However, our results suggest that autonomous selfing can set as many fruit and seed as natural pollination, indicating autonomous selfing as the main contrivance of natural pollination in *R*. *alpina*. Our observations indicate that in a majority of flowers there is gradual shrinkage of style from the second day of anthesis. On the third day of flowering, the stigma is completely overtopped by the surrounding anther lobes making clear contact between anther and stigma. Our data suggest that shrinkage of style occurs at ca 2mm to allow self-pollination. Thus, *R*. *alpina* despite clear spatial separation between anther and stigma during the initial hours of flowering, undergoes autonomous selfing by the shrinkage of style and ensure the reproductive success if no pollinator vector has already provided effective pollen transfer. Our results also indicate that majority of autonomous selfing occurs on the third day of anthesis while there was no autonomous selfing on the first day of anthesis which further supports that autonomous selfing occurs by the gradual shrinkage of style on the succeeding days of anthesis. This pollination mechanism is partly consistent with the pollination mechanism in *R*. *scheneideria* which achieves autonomous selfing by the curling of hooked stigmas towards the dehisced anthers [40] and *R*. *debilis* which assures reproductive success via autonomous selfing aided by the secretion of stigmatic fluids [33]. We conclude that the evolution of autonomous selfing in *R*. *alpina*, the highest elevational ginger on earth [24], is one of the evolutionary strategies to assure the reproductive success in a zone of unreliable pollinator service, which is similar to pollination mechanisms in some other alpine plants [41].

### Beetle as an obligate pollinator

Beetle-pollinated flowers are typically characterized by the presence of discernible fragrance that acts as a primary attractant [42]. Other reported features of beetle-pollinated flowers include either sufficient nutritional rewards (mostly pollen grains and sometimes special nutritive tissue) or protection from predators [43,44]. Here we reported the evidence of beetle pollination system in a small alpine ginger, *R*. *alpina*, which lacks cantharophily syndromes. Like other members of the genus, *R*. *alpina* also exhibits strikingly long tongued fly pollination syndromes. Somewhat surprisingly however, we observed a beetle as the exclusively observed legitimate pollinator of this small alpine ginger in the Himalaya of Nepal. Our extensive observation across the study site that encompasses more than 5000 flowering individuals indicated that the only other visitor to the flowers of *R*. *alpina* were rare visits by the moth (*Macroglossum nyctersis*). Although, we recorded very few visits of the moth to the flowers of *R*. *alpina*, it never made legitimate contact with the reproductive parts of the flower. Moreover, our data also suggest that moth visited flowers neither received any pollen grains on their stigma nor set any fruit/seed. Thus the moth is a nectar robber of *R*. *alpina* rather than a potential pollinator. Despite the apparent absence of cantharophily syndromes, it is possible that the unelaborated and open construction of flower in *R*. *alpina* may have provided easy access to the beetle as in other beetle pollinated flowers [45]. Although more than 184 angiosperm species from 34 families are known to be almost exclusively pollinated by beetles [10], to the best of our knowledge, this is the first direct evidence of beetle pollination system in Zingiberaceae, in which all other known members are found to be pollinated by long distance foragers such as bees, birds and/or flies [18,22,23,46].

Given the new finding that *Mylabris sp*. is the sole observed pollinator of *R*. *alpina* in the Nepalese Himalaya, it is interesting to consider how these beetles may orientate to the flowers. Whilst beetle pollination is classically thought to be mediated by scent [10], flowers of this genus are associated with having an absence of fragrance [24] and so vision is a likely perceptual channel for finding flowers. Within Coleoptera it is known that species can have two (dichromatic), three (trichromatic) or four (tetrachromatic) photoreceptor type input to their visual system to potentially facilitate colour vision [47,48], and recent work on the maize weevil *Sitophilus zeamais* beetle, which is classified as a pest, indicates an innate orientation in some adult beetles mediated by colour visual information [49]. Interestingly, whilst previous work on blister beetles, *Hycleus* spp. (Coleoptera: Meloidae) suggests they may damage flowers [50,51], we did not observe this at our sites, and indeed the data suggests these beetles were effective pollinators of *R*. *alpina*. Coloured trap experiments with some flower visiting beetles suggest that colour is used in a functional way that has driven the evolution of flower colouration [12,52]. In the Mediterranean region beetle pollinated flowers are often red, which fits with evidence of a trichromatic and long wavelength sensitive visual system from the flower beetle (*Pygopleurus israelitus*) of that region [12,52,53]. *Pygopleurus israelitus* is of family Glaphyridae of suborder polyphaga and infraorder Elateroidae for which there have been several reports of trichromacy [53–55]; however, the *Trilobium castaneum* superfamily of Tenebrionidae of order polyphaga most likely only possess dichromatic visual systems [56]. The Nepalese *Mylabris sp*. in the current study belongs to family Meloidae of infraorder Cucujiformia; and also belongs to same superfamily Tanebrionidae, thus dichromatic vision would be a plausible visual system for these beetles, although the diversity of colour vision within beetle pollinators may vary spatially [47,48,53]. The pollen beetle *Meligethes aeneus* f*abricius* (Coleoptera, Nitidulidae) appears to also conform to dichromatic vision potentially enabling colour choices within Tanebrionidae [57]. Using the principle outlined by Kemp et al.[34] of using nearest relevant phylogenetic model to test colour theories on signal-receiver relationships, future work could explore how *Mylabris* sp. may view flowers considering the likely UV (330-360nm peak range) and ‘Green’ (530-560nm) dichromatic vision [47] within the Tanebrionidae superfamily, and we provide spectra data of *R*. *alpina* to facilitate this. Indeed, the spectral data in Fig 2 does not have characteristics of ‘red’ flowers which should possess a sharp change in reflectance of greater than 20% over a 50nm region of the spectrum [36,37] as have been associated with bird pollinated red flowers [58] and suggested for beetle pollinated red flowers based upon colour categories [12]. The flower spectra possess a sharp change in spectral reflectance for wavelengths less than 420nm; which was not a characteristic of the flowers of Macquarie Island where pollination appears to be mediated by flies [35]. The flower spectral is also unusual in that it reflects a lot of UV and longer wavelength radiation, a characteristic that is not consistent with bee pollinated flowers [59–61], but the strong change in reflectance is indicative of an evolved signal to a pollinator [58,62].

## Conclusions

We show that, different to classic pollination syndromes, *R*. *alpina* employs dual pollination mechanisms to help maximize its reproductive success in a sub-alpine zone of unreliable pollinator service. Our results suggest that the evolution of autonomous selfing as a predominant mode of reproduction coupled with beetle pollination system in *R*. *alpina* provide reproductive assurance to this alpine ginger. This result provides the first experimental evidence of beetle pollination system in Zingiberaceae. Indeed, the involvement of a beetle (*Mylabris sp*.) as the only pollinator of *R*. *alpina* suggests that a novel type of plant-pollinator interaction may be present to what has previously been considered for beetle pollination, and indicates that beetles play an important role for improving the genetic diversity of *R*. *alpina* in the Nepalese Himalayas. Flower spectra for *R*. *alpina* is different to ‘red’ signals classically associated with beetle vision, suggesting a different type of visual processing may operate in this plant-pollinator interaction at high altitudes.

## Supporting information

S1 Table Variation in the visitation frequency of *Mylabris* species across three plots for two years. Result analyzed with two way ANOVA showing the effect of years and plots on visitation frequency of a beetle (*Mylabris sp*.) to the flowers of *R*. *alpina*.
(DOCX)

S2 Table Test of capacity of autonomous selfing for the natural breeding of *R*. *alpina*. Result of generalized linear model to examine the difference in fruit set percentage and seed number per fruit between autonomous self-pollination and natural pollination in the flowers of *R*. *alpina* in 2015 and 2016.
(DOCX)

S3 Table Test of contribution of pollinators for the natural breeding of *R*. *alpina*. Result of generalized linear model to examine the difference in fruit set percentage and seed number per fruit between emasculated-open and naturally pollinated flowers of *R*. *alpina* in 2015 and 2016.
(DOCX)

S4 Table Test of self-compatibility in *R*. *alpina*. Result of generalized linear model to examine the difference in fruit set percentage and seed number per fruit between hand self-pollinated and hand cross pollinated flowers of *R*. *alpina* in 2015 and 2016.
(DOCX)

S5 Table Test of pollination efficiency of a beetle (*Mylabris sp*.) for the natural breeding of *R*. *alpina*. Result of generalized linear model to examine the difference in fruit set percentage and seed number per fruit among natural pollination, autonomous selfing and emasculated-beetle pollinated flowers of *R*. *alpina* in 2015 and 2016.
(DOCX)
