# Supplementary material for: Ginger and the beetle: Evidence of primitive pollination system in a Himalayan endemic alpine ginger (Roscoea alpina, Zingiberaceae)
Source: PLoS One. 2017 Jul 19;12(7):e0180460. doi: 10.1371/journal.pone.0180460 (PMC5516977; doi:10.1371/journal.pone.0180460)
Supplement: S1 Table — Result analyzed with two way ANOVA showing the effect of years and plots on visitation frequency of a beetle (Mylabris sp.) to the flowers of R. alpina. (DOCX) [file pone.0180460.s001.docx]

# Supporting Information- S1 Table (Manuscript number-PONE-D-17-05192)

# Ginger and the beetle: evidence of primitive pollination system in a Himalayan endemic alpine ginger (*Roscoea alpina*, Zingiberaceae)

Babu Ram Paudel^1, 2, 3^, Mani Shrestha^4, 5^, Adrian G. Dyer^4^ and Qing-Jun Li^6^**^*^**

^1^Key laboratory of Tropical Forest Ecology, Xishuangbanna Tropical Botanical Garden,

Chinese Academy of Sciences, Yunnan, 666303, China

^2^University of Chinese Academy of Sciences, Beijing, 100039, China

^3^Department of Botany, Prithvi Narayan Campus, Tribhuvan University, Pokhara, Nepal

^4^School of Media and Communication, RMIT University, Melbourne, Victoria, 3001, Australia

^5^Faculty of Information Technology, Monash University, Melbourne, Victoria, 3800, Australia

^6^Laboratory of Ecology and Evolutionary Biology, State Key Laboratory for Conservation and Utilization of Bio-Resources in Yunnan, Yunnan University, Kunming, Yunnan, China

***** For correspondence: [qingjun.li@ynu.edu.cn](mailto:qingjun.li@ynu.edu.cn) (QJL)

**S1 Table. Variation in the visitation frequency of *Mylabris* species across three plots for two years**. Result analyzed with two way ANOVA showing the effect of years and plots on visitation frequency of a beetle (*Mylabris* *sp.*) to the flowers of *R. alpina.*

| Source | Sum of Squares | df | Mean Square | F | P |
| --- | --- | --- | --- | --- | --- |
| Years | 0.001 | 1 | 0.001 | 0.021 | 0.885 |
| Sites | 0.001 | 2 | 0 | 0.012 | 0.989 |
| Years x sites | 0.006 | 2 | 0.003 | 0.109 | 0.897 |
| Error | 1.702 | 66 | 0.026 |  |  |
