# Supplementary material for: Ginger and the beetle: Evidence of primitive pollination system in a Himalayan endemic alpine ginger (Roscoea alpina, Zingiberaceae)
Source: PLoS One. 2017 Jul 19;12(7):e0180460. doi: 10.1371/journal.pone.0180460 (PMC5516977; doi:10.1371/journal.pone.0180460)
Supplement: S2 Table — Result of generalized linear model to examine the difference in fruit set percentage and seed number per fruit between autonomous self-pollination and natural pollination in the flowers of R. alpina in 2015 and 2016. (DOCX) [file pone.0180460.s002.docx]

# Supporting Information- S2 Table (Manuscript number-PONE-D-17-05192)

# Ginger and the beetle: evidence of primitive pollination system in a Himalayan endemic alpine ginger (*Roscoea alpina*, Zingiberaceae)

Babu Ram Paudel^1, 2, 3^, Mani Shrestha^4, 5^, Adrian G. Dyer^4^ and Qing-Jun Li^6^**^*^**

^1^Key laboratory of Tropical Forest Ecology, Xishuangbanna Tropical Botanical Garden,

Chinese Academy of Sciences, Yunnan, 666303, China

^2^University of Chinese Academy of Sciences, Beijing, 100039, China

^3^Department of Botany, Prithvi Narayan Campus, Tribhuvan University, Pokhara, Nepal

^4^School of Media and Communication, RMIT University, Melbourne, Victoria, 3001, Australia

^5^Faculty of Information Technology, Monash University, Melbourne, Victoria, 3800, Australia

^6^Laboratory of Ecology and Evolutionary Biology, State Key Laboratory for Conservation and Utilization of Bio-Resources in Yunnan, Yunnan University, Kunming, Yunnan, China

***** For correspondence: [qingjun.li@ynu.edu.cn](mailto:qingjun.li@ynu.edu.cn) (QJL)

**S2 Table. Test of capacity of autonomous selfing for the natural breeding of *R. alpina*.** Result of generalized linear model to examine the difference in fruit set percentage and seed number per fruit between autonomous self-pollination and natural pollination in the flowers of *R. alpina* in 2015 and 2016.

|  | Fruit set percentage | | | Seed number per fruit | | |
| --- | --- | --- | --- | --- | --- | --- |
|  | df | deviance | P value | df | deviance | P value |
| Year (Y) | 1 | -0.5423 | 0.375 | 1 | -0.06441 | **0.0376** |
| Treatment (T) | 1 | -0.9150 | 0.131 | 1 | 0.01021 | 0.7539 |
| Y x T | 1 | 0.8562 | 0.303 | 1 | -0.01046 | 0.8233 |
